# Body Stalk Anomaly in a 9-Week Pregnancy

**DOI:** 10.1155/2014/357285

**Published:** 2014-05-29

**Authors:** Fabio E. Quijano, María Mónica Rey, Mariana Echeverry, Roland Axt-Fliedner

**Affiliations:** ^1^Department of Gynecology, Obstetrics and Human Reproduction, Fundación Santa Fe de Bogotá Hospital, Bogotá, Colombia; ^2^Department of Gynecology, Obstetrics and Human Reproduction, Fundación Santa Fe de Bogotá Hospital, Universidad de los Andes, Bogotá, Colombia; ^3^Maternal Fetal Unit, Giessen, Germany

## Abstract

Body stalk anomaly is a rare and severe malformation syndrome in which the exact pathophysiology and trigger factors are still unknown. This is a case of a 30-year-old patient who underwent ultrasound at 9 weeks of gestation. It revealed an abnormal location of the inferior body of the embryo in the coelomic space. The findings suggested a short umbilical cord syndrome. In order to confirm the diagnosis, the patient was scheduled for a second ultrasonography at 11 weeks of gestation. The obtained images, confirmed the location of the inferior body in the coelomic space with no visible bladder, absence of the right leg, severe abdominal wall defect, consistent with an omphalocele, and a short 5 mm umbilical cord. These last ultrasonographic findings were consistent with body stalk anomaly. Because of severe malformation incompatible with life, the patient was offered termination of pregnancy. Pathologic examination confirmed the suspected pathology of body stalk anomaly.

## 1. Introduction

Body stalk anomaly is a severe defect of the abdominal wall in which there is evisceration of abdominal organs and in more severe cases of thoracic organs as well. This congenital malformation is accompanied by severe kyphoscoliosis and the presence of a rudimentary umbilical cord which is usually short or even absent [1]. Likewise, this anomaly might also occur in conjunction with neural tube defects, genitourinary malformations, abnormalities of the chest wall, intestinal atresia, and craniofacial defects, among others [2, 3]. The variety of phenotypes in the reported cases worldwide has led to the creation of a confusing array of terms for this condition including the amniotic band syndrome, short umbilical cord syndrome, and limb-body-wall complex [4]. This rare malformation syndrome has an estimated incidence of 1/14.000 to 1/31.000 pregnancies [5, 6]. However, in a most recent multicenter study of Daskalakis et al., in which 106,727 fetuses between 10 and 14 weeks of gestation were analyzed, an incidence of 1/7,500 pregnancies was found [1]. This great discrepancy in the incidence rates suggests that this type of malformation might be responsible for a significant number of spontaneous abortions during the first trimester of pregnancy, and thus the real incidence for this anomaly might be underestimated.

When it comes to pathogenesis, different theories have been proposed to explain the possible mechanism by which this anomaly is triggered. The actual mechanism, however, still remains unclear. In most of the described cases, the karyotypes of the affected fetuses have been completely normal, and only in two cases there have been chromosomal abnormalities associated with uniparental disomy of chromosome 16 [7] and with a trisomy of chromosome 2. This latter is probably due to a confined placental mosaicism. Hence, what is really known about the defects of the body stalk is that there are environmental and genetic factors that play an important role in the pathophysiology of this complex and poorly understood condition [8].

## 2. Case Report

We report a case of a 30-year-old German woman who was first seen by the maternal-fetal medicine service for her first prenatal visit at 5 weeks of gestation at the Hospital Universitario Santa Fe de Bogota. This was her first gestation, and she had a history of recurrent urinary tract infections that required intravenous antibiotics. She was receiving a folic acid supplement and progesterone, and there was not any other relevant past medical or surgical. Her initial prenatal labs were within the normal limits.

**Figure 1:**
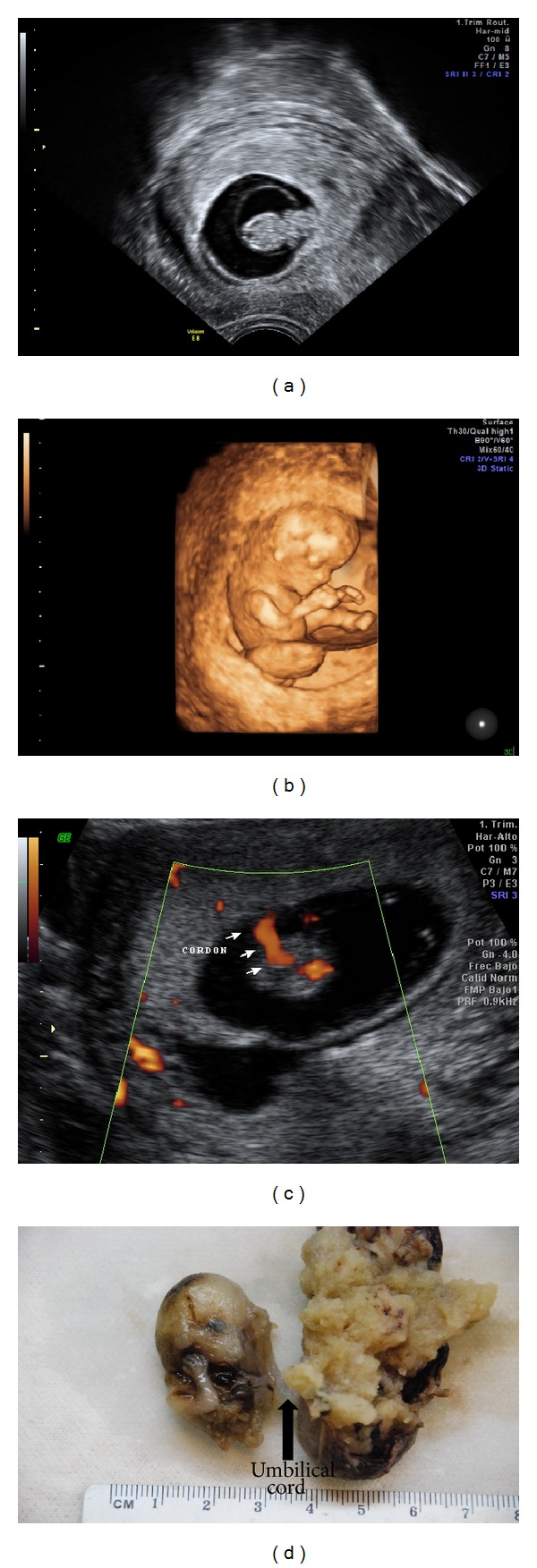
(a) The inferior body in the coelomic space. (b) Absence of the right leg. (c) Short umbilical cord of 5 mm. (d) Defect of the anterior and lower abdominal wall, with exposure of the intestine, liver, and spleen. The left leg was folded into the trunk. The upper extremities were normal.

At 9 weeks of gestation, she had her first ultrasound examination which revealed a normal fetal crown-rump length of 2, 13 cm and an abnormal location of the inferior body of the embryo, in the coelomic space (Figure 1(a)). These findings suggested a short umbilical cord syndrome. In order to confirm the diagnosis the patient was scheduled for a second ultrasound at 11 weeks of gestation. The obtained images confirmed the location of the inferior body in the coelomic space, with no visible bladder, absence of the right leg (Figure 1(b)), a severe abdominal wall defect, compatible with an omphalocele, and a short umbilical cord of 5 mm (Figure 1(c)). These last ultrasonographic findings were consistent with body stalk anomaly.

Because the malformation was considered to be incompatible with life, patient was offered to undergo voluntary termination of pregnancy. The embryo was sent to pathology after PG-induction. The embryo showed gross morphologic characteristics as follows: normal conformation of head with normal upper limbs, absence of the right leg, left leg bent toward the chest, and a severe omphalocele containing the bowels and liver.

The placenta had a measurement of 5 × 4 cm. Fetal umbilical cord was identified anteriorly, with central insertion and a short length of 2 cm and 0.3 cm diameter, wrapped by amniotic membranes that trapped the fetus. The fetus was immature and malformed. Head, part of the trunk, upper extremities, and left lower extremity were recognized. The face was symmetrical and the nose was flattened and lowered. The upper lip was not recognized, the lower jaw was maintained, and eyes had hypotelorism. The external ear structures were rudimentary and with low implantation. The trunk had scoliosis. There was a defect of the anterior and lower abdominal wall, with exposure of the intestine, liver, and spleen. The left leg was folded into the trunk. The upper extremities were normal. Findings were compatible with limb-body-wall complex (Figure 1(d)). The microscopic examination revealed immature chorionic villi that corresponded to the first trimester, with erythroid and nucleated forms. The membranes and the decidua were unaltered, and the fetal tissue was in continuity with amniotic membranes. The cytogenetic studies did not reveal numeric alterations in chromosomes 13, 18, and 21.

## 3. Discussion

Body stalk anomaly is a term used to describe a pattern of severe defects that in most of the reported cases proves to be incompatible with life. As described above, this condition should be suspected when a large abdominal defect is observed as well as abnormalities in the axial skeleton such as kyphosis or scoliosis, and a short or absent umbilical cord is present [9]. Body stalk defects can be detected at the end of the first trimester of pregnancy by ultrasound. In our case, the detection was done at 9 weeks of gestation and then reconfirmed at 11 weeks. The ultrasonographic findings were consistent with those reported in the literature. Some authors suggest that this anomaly might be detected by measuring the levels of alpha-fetoprotein in maternal serum, mainly in the second trimester of pregnancy, in which high levels of this marker have been reported to detect 100% of cases of this anomaly [6]. Nonetheless, it is essential to make an early diagnosis in order to provide the future parents with the necessary information and counseling regarding the prognosis of this type of anomaly, by making emphasis on the benefits of early pregnancy termination and taking into account the possible complications that can arise during childbirth and/or pregnancy, as well as on the fact that this type of anomaly is lethal in most of the cases. It is also important to recall that there are no specific therapeutic interventions for the fetus that usually dies shortly after its delivery [8].

An appropriate midsagittal view of the fetus for the measurement of the crown-rump length, together with the measurement of the nuchal translucency and adequate sweeps through the head and abdomen, should identify all the cases of body stalk anomaly between 11 and 13 weeks of gestation [10]. Before establishing a final diagnosis, it is important to consider other pathologies that affect the abdominal wall such as omphalocele, gastroschisis, vesical exstrophy, Cantrell pentalogy, amniotic band syndrome, Beckwith-Wiedemann Syndrome, and the OEIS complex (omphalocele, exstrophy of cloaca, imperforate anus, and spinal defects) [11].

In our case report, we did not observe any significant risk factor associated with the environmental exposure to teratogens. However, in some case series, it has been reported that 50% of women with fetuses affected by body stalk anomaly smoke cigarettes or drink alcohol and 30% of them smoked marijuana [12].

Three major theories have been proposed to explain the origin of the main phenotypic features that are found in body stalk anomaly. The first and most accepted theory is an early rupture of the amnion before there is an obliteration of the coelom. Another theory proposed by Steeter et al. suggests an abnormal folding of the trilaminar disc in its cephalic, caudal, and lateral directions that might lead to the persistence of the coelomic cavity [13]. Finally, Van Allen et al. proposed that a vascular compromise in early gestational weeks might be the cause of an inadequate closure of the abdominal wall as well as the cause of instability of the amnion which then undergoes an early rupture [13].

Body stalk anomaly is a malformation syndrome in which the exact pathophysiology and trigger factors are still unknown. Much remains to be elucidated in terms of its real epidemiology, global distribution, and risk factors. Additionally efforts should focus on making an early diagnosis in order to avoid complications for the mother during the pregnancy and/or childbirth. Although most of the reported cases in the literature suggest that an early diagnosis can be made between 10 and 14 weeks of gestation, in our case the diagnosis was suspected earlier, at nine weeks of pregnancy. This turns out to be beneficial for both the expecting parents and the physician, as it enables a prompt knowledge of the fetus condition that permits giving an appropriate counseling and a timely management.
